# Iliac Artery Occlusion Following Iliac Vein Recanalization and Stenting in Two Patients

**DOI:** 10.1177/15385744251327018

**Published:** 2025-03-21

**Authors:** Thomas Pennix, Zayed Metwalli, Peiman Habibollahi

**Affiliations:** 1 UT Health Science Center at Houston, Houston, TX, USA; 2 MD Anderson Cancer Center, Houston, TX, USA

**Keywords:** endovascular techniques, endovascular device, vascular imaging

## Abstract

Ileocaval venous thrombosis and outflow obstruction is a condition with many possible causes that typically presents with symptoms related to venous congestion or insufficiency. Recent device development and availability of endovascular stenting and venoplasty for lifestyle-limiting symptoms refractory to conservative management has led to increased interest in these procedures. While several common complications of venous stent placement have been well-described, 1 uncommon and emergent complication is nearby arterial compression or occlusion. Here we present 2 cases of iliac artery occlusion occurring after iliac venous recanalization and stenting, and discuss possible factors that may contribute to this uncommon complication.

## Introduction

Iliocaval venous thrombosis and outflow obstruction may occur secondary to many etiologies, such as underlying hypercoagulability or extrinsic compression of the venous system. This condition typically presents symptoms of venous insufficiency and increased venous pressure, such as lower extremity edema or ulceration. Conservative management includes the use of compression stockings, exercise, and local wound care. Lifestyle-limiting obstructive venous disease refractory to conservative management may be treated with percutaneous stenting and venoplasty. Although several common complications of this procedure have been well-described such as stent thrombosis or migration, an additional rare complication to consider is arterial compression following venous stent placement.^1^ This is an uncommon complication of venous stent placement, with only a few prior reported cases.^2-7^ Hypothesized causes for arterial occlusion following venous stenting include regional cancer, prior surgery and radiation, and altered anatomy such as May-Thurner’s amongst others. Here we present 2 cases of iliac artery occlusion occurring after iliac vein recanalization and stenting.

## Case 1

**Figure 1.**
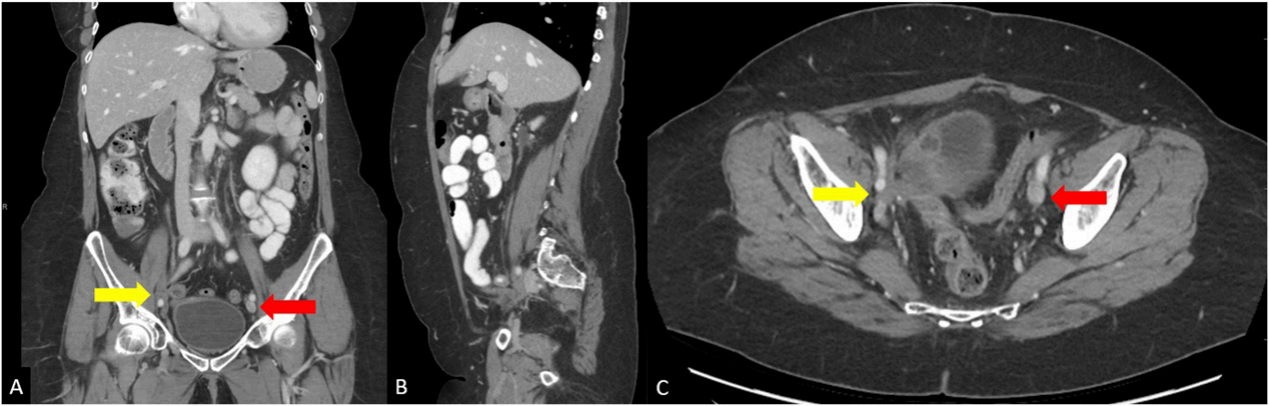
(A) Coronal, (B) sagittal, and (C) axial CT occlusion of the right external iliac vein with nearby soft tissue pelvic mass (yellow arrow). Normal opacification of the left external iliac vein can be seen (red arrow).

The first patient is a 49-year-old female with a history of stage IV cervical cancer and DVT’s on lifelong anticoagulation with therapeutic lovenox who presented with 2 years of right leg pain and swelling refractory to compression stockings and exercise. She had previously undergone total hysterectomy with salpingo-oopherectomy and pelvic radiation therapy. Physical exam showed pitting edema in the right lower extremity with palpable pulses, and normal exam of the left lower extremity. Initial CT imaging was significant for pelvic metastases and occlusion of the right external iliac vein (Figure 1).

The patient was taken for venogram, which showed tandem occlusions of the right mid-femoral vein and right external iliac vein, with extension into the proximal common femoral vein and distal common iliac vein. Following traversal of the occlusions, balloon angioplasty of the stenotic segments was performed.

**Figure 2.**
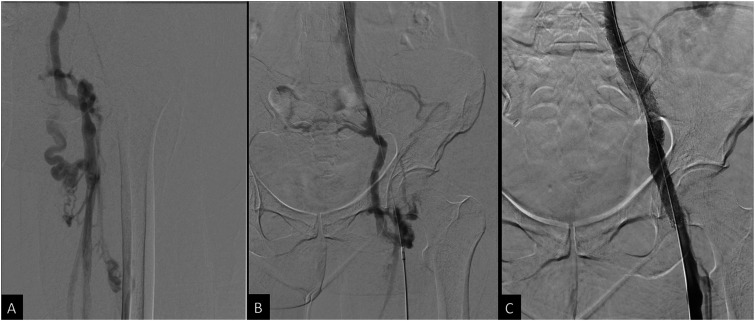
(A) and (B) Initial venogram showed tandem occlusions of the right mid-femoral vein and right external iliac vein, with extension into the proximal common femoral vein and distal common iliac vein. (C) Completion venogram showing restoration of flow throughout the right iliofemoral system without collateral opacification.

Intravascular ultrasound (IVUS) was then used for optimal stent sizing and positioning at the site of compression by the treated pelvic tumor. A 14 mm × 14 cm self-expanding nitinol stent (Zilver Vena, Cook Medical, Bloomington, Indiana, USA) was deployed within the stenotic external iliac/common femoral vein. The stent was subsequently post-dilated using 12 mm and 14 mm high pressure angioplasty balloons. Completion venogram showed satisfactory restoration of flow throughout the right femoral, common femoral, and iliac veins without significant residual stenosis or persistent venous collateral opacification (Figure 2).

**Figure 3.**
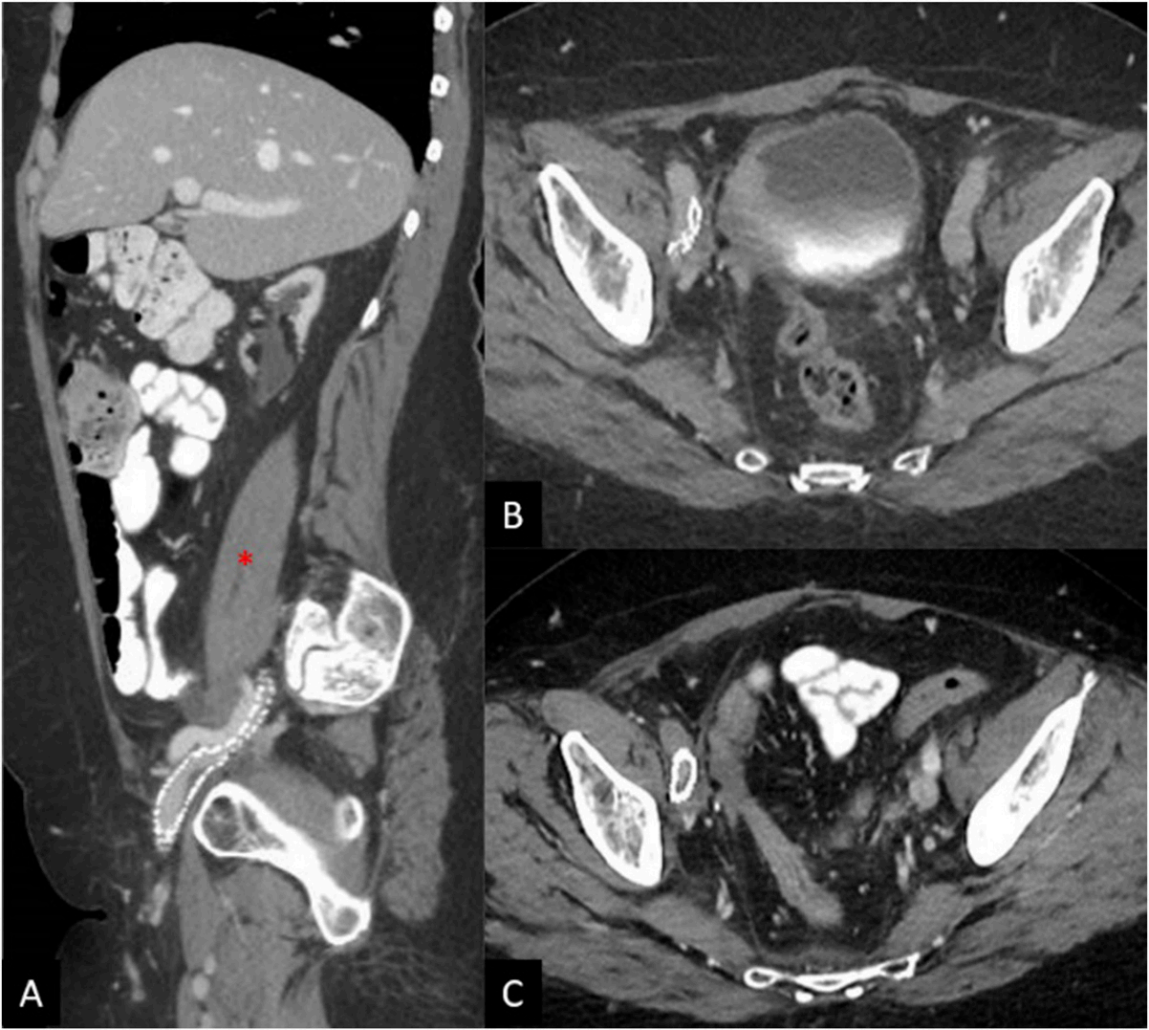
(A) Sagittal CT image shows compression of the stent in the external iliac vein. Pelvic tumor (*) is anterior to the right external iliac artery. (B) Axial CT image shows stent recoil with compression of the stent at the level of the right external iliac artery. (C) Axial CT image after balloon expandable stent placement in the right external iliac vein shows persistent, but improved stent compression. The right external iliac artery is now occluded with distal reconstitution of the common femoral artery (not shown).

**Figure 4.**
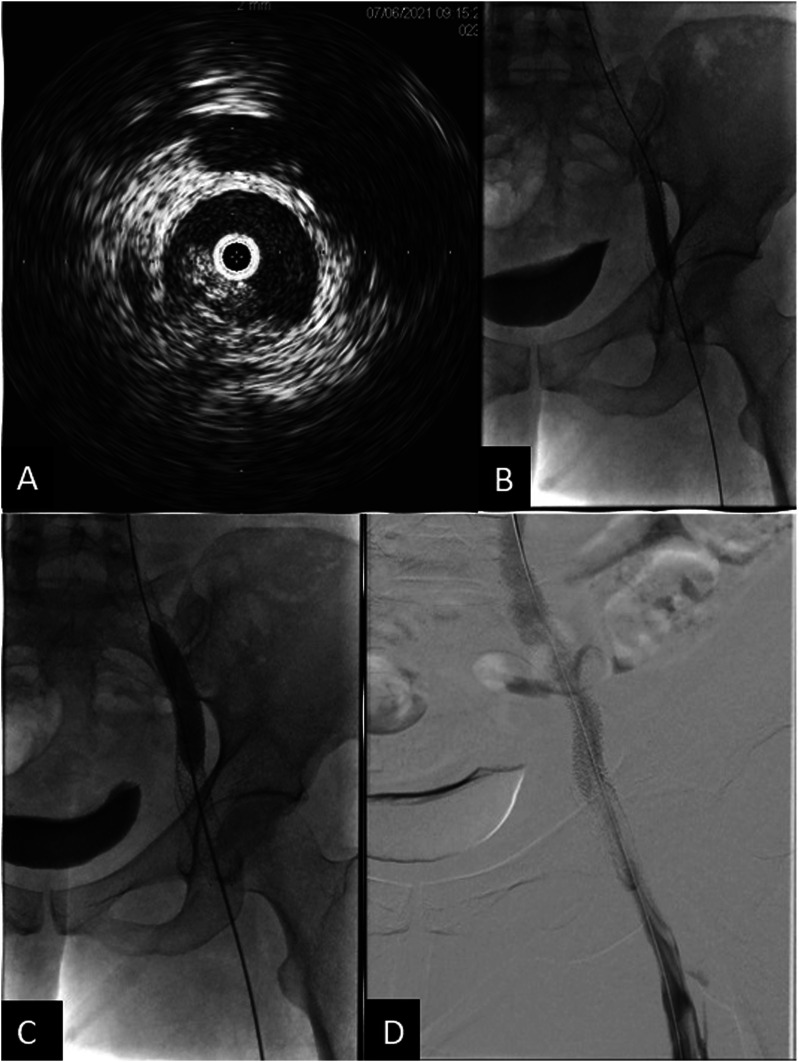
(A) IVUS showing high-grade focal stenosis within the mid to distal aspect of the right external iliac vein stent. (B) 8 × 59 mm VBX Balloon expandable covered stent successfully deployed at the site of the high-grade stenosis. (C) Progressive dilation of the stent using 10 × 60 mm and 12 × 40 mm balloon catheters. (D) Final venogram showed patent right femoral vein and the stented right external iliac vein with brisk emptying of contrast and no significant collateral venous opacification.

The patient noted mildly improved symptoms soon after the procedure, but experienced increased swelling and pain 2 months later. Follow up CT demonstrated stent recoil and compression by the pelvic metastasis (Figure 3A, and B). A subsequent venogram and IVUS showed recurrent occlusion of the right femoral vein, as well as high-grade stenosis in the mid portion of the previously placed right external iliac vein stent due to external compression. Angioplasty of the right femoral vein occlusion/stenosis was performed, and an 8 × 59 mm balloon-expandable covered stent (VBX, Gore Medical, Newark, Delaware, USA) was deployed within the previously placed stent. Repeat IVUS showed appropriate expansion without residual stenosis or significant stent recoil, but persistent irregularity and stenosis of the recanalized right femoral vein segment, which was treated with repeat prolonged balloon angioplasty. The final venogram demonstrated patency of the right femoral vein and stents (Figure 4). Her anticoagulation regimen was continued postoperatively.

At follow up 2 weeks later, the patient endorsed improvement in right leg swelling, but decreased warmth and new calf pain with activity, consistent with claudication without rest pain or soft tissue ulceration. Pulses remained dopplerable. Contrast-enhanced CT imaging revealed improved narrowing of the right external iliac vein, but new thrombosis of the right external iliac artery with reconstitution of the common femoral artery (Figure 3C). Prior to venous recanalization, the right external iliac artery had been patent with only moderate non-flow-limiting stenosis. Right ankle brachial index (ABI) was 0.69, consistent with moderate-severe arterial occlusive disease, and toe brachial index (TBI) was absent. After vascular surgical consultation, conservative management with cilostazol was suggested with subsequent improvement in claudication. Given the lack of soft tissue ulceration and lack of symptom severity, invasive arterial intervention was not indicated. Furthermore, her symptoms resolved with conservative management and arterial revascularization risked worsening symptoms of deep venous insufficiency, as stenting of the external iliac artery would likely compress the adjacent venous stent.

At follow-up 3 months later, the patient noted continued improvement in lower extremity swelling and claudication, with repeat right-sided ABI showing 0.69, consistent with only moderate right leg occlusive disease, and TBI showing 0.63, consistent with mild occlusive disease. The patient was followed for an additional 2 years without recurrence of claudication. The right external iliac venous stent remained patent at last follow-up and patient was maintained on anticoagulation therapy.

## Case 2

**Figure 5.**
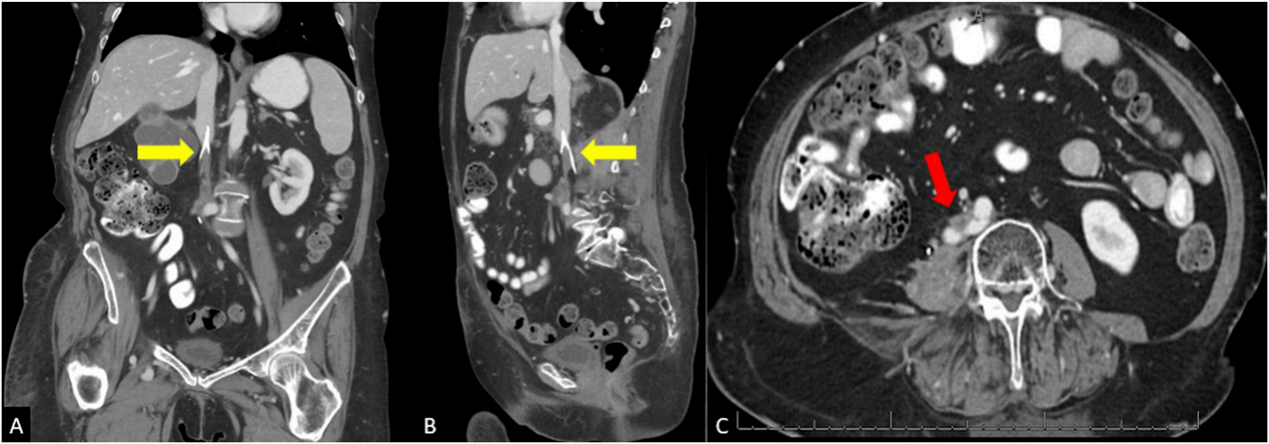
(A) Coronal and sagittal (B) CT shows chronic occlusion of the IVC with pre-existing filter (yellow arrow). (B) Axial images slightly lower show compression of the right common iliac by a mass prior to venous stenting (red arrow).

The second patient is a 67-year-old female with a history of chronic pulmonary embolisms and DVT’s on lifelong anticoagulation with apixaban with previously placed IVC filter, obstructive CKD, and stage IV endometrial cancer who presented with several months of progressive bilateral lower extremity swelling greatest on the right. CT scan from 1 month prior showed chronic thrombosis of the IVC below a previously placed filter, with large metastases in the retroperitoneum mildly compressing the right common iliac artery (Figure 5).

**Figure 6.**
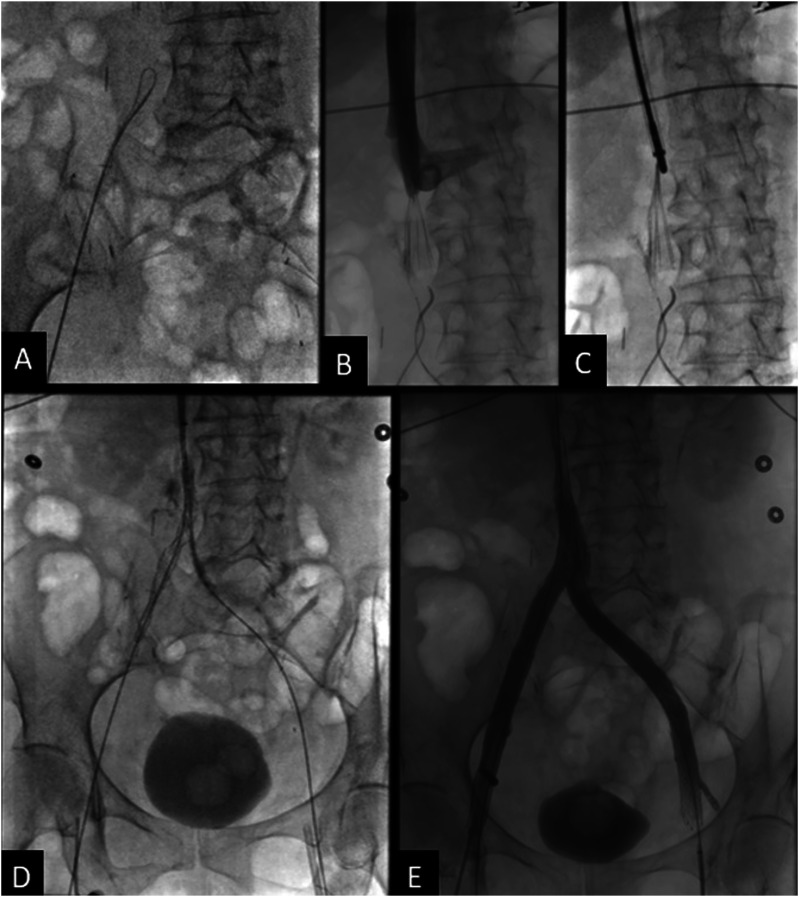
(A) Venous recanalization was performed using multiple catheters, wires, and crossing catheters from both the common femoral veins and right internal jugular vein. (B) Venogram shows thrombosis of the IVC inferior to the filter. (C) Filter removed via forceps through in its entirety. (D) Stenting of infrarenal IVC and bilateral common iliacs using 14 mm Abre stents in Y configuration followed by placing 12 mm stents in the external iliac veins. All the stents were plastied to 10 mm and 12 mm eventually. A total of 3 14 mm Abre and 2 12 mm Abre stents were used. (E) Repeat venogram at the end of the procedure showed satisfactory patent bilateral iliacs and infrarenalcava with good forward flow.

Bilateral femoral venograms showed chronic occlusion and marked collateralization of flow from both sides. Venous recanalization was performed, and the filter was retrieved in its entirety via forceps and sheath. Subsequent venogram demonstrated no damage or extravasation. The bilateral common iliac veins and infrarenal IVC were then stented using 14 mm self-expanding (Abre, Medtronic, Minneapolis, Minnesota, USA) stents in Y configuration, followed by placement of 12 mm stents in the external iliac veins. Final venogram showed patency of the bilateral iliacs and infrarenal cava with good forward flow (Figure 6).

**Figure 7.**
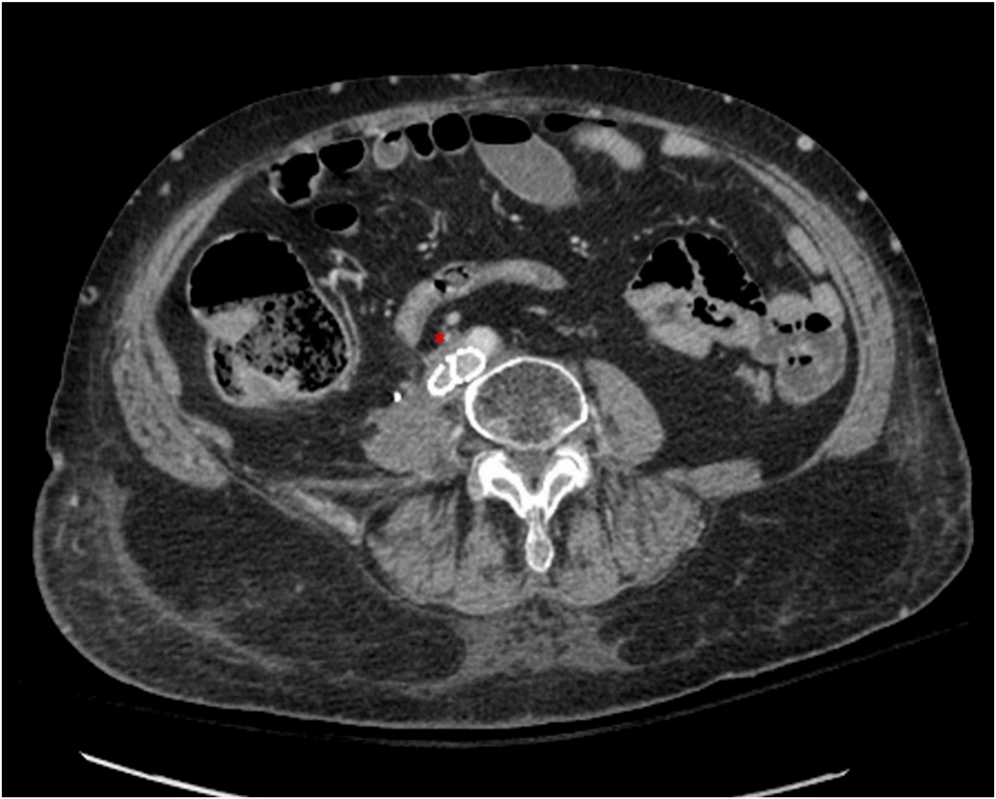
Axial CTA in venous phase shows compression of the right external iliac artery by the stent, and narrowing of the stent.

On postoperative day 1, the patient endorsed right leg pain which was initially managed conservatively. Overnight, the pain increased along with developing numbness, decreased warmth, and absent doppler pulses. CTA showed stenosis of the IVC and left external iliac stents, with compression of the right common iliac artery by the venous stent (Figure 7).

**Figure 8.**
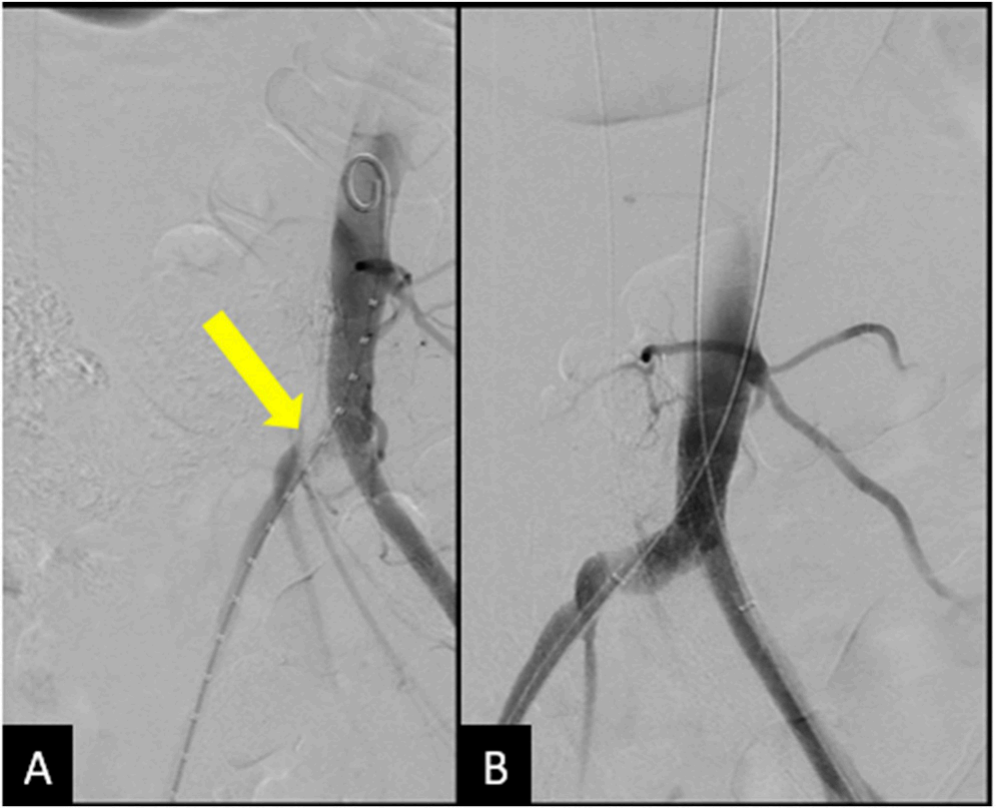
(A) Angiogram showing compression of the right external iliac artery at the level of the iliac vein stent. And performed an angiogram showing the occlusion. (B) Angiogram following placement of 8 mm VBX stents deployed in a kissing fashion, showing patency and brisk flow through bilateral iliofemoral systems with no injury.

Vascular surgery was consulted and performed an angiogram, which showed occlusion of the right common iliac artery secondary to compression by the venous stent. 8 mm VBX stents were deployed in a kissing fashion across the external iliac arteries bilaterally, with subsequent angiogram showing patency of the bilateral iliofemoral systems and right leg, which had palpable pulses at the end of the procedure (Figure 8).

The patient was transferred to the SICU for vascular monitoring. On Postoperative day 1, her right lower extremity symptoms had resolved with palpable pulses. She was transferred to the floor and underwent successful additional stenting of the stenosed IVC and left external iliac stents. Her admission was extended for workup of chronic thrombocytopenia requiring bone biopsy, and she was discharged on postoperative day 7 after stent revision. Her oral anticoagulation regimen was continued postoperatively. ABI’s 2 weeks later were normal bilaterally, and stents were patent on 1 month follow up CT. At a clinic visit 3 months later, the patient noted markedly improved swelling bilaterally.

## Discussion

The increasing utilization of endovascular stenting for the treatment of severe venous obstruction requires awareness of patient-specific factors which may complicate the procedure or effect its long-term success. These 2 cases highlight this importance while providing several important considerations for future research and physicians performing the procedure. First, both patients had intrapelvic masses with histories of prior pelvic surgery and radiation, which is shared with almost all the reported cases.^2-5,7^ Compression from nearby masses combined with the fibrotic effects of prior radiation and surgery are likely strong predisposing factors to arterial compression following venous stent placement, as there is decreased elasticity of the surrounding soft tissues.

As noted by Elsayed et al, the external iliac artery seems to be the most commonly occluded, which is true for our first case and likely secondary due to its smaller size and tighter surroundings compared to the larger common iliac artery. Similar to Taylor et al, however, the right common iliac artery was occluded in our second case. This may be due to the more proximal stenosis in both cases, with chronic occlusion of the distal IVC in our second case and clot extension into the distal IVC in the case reported by Taylor et al. Additionally, both patients had regional mass effect, with pre-existing mild compression of the right common iliac artery in our case and retroperitoneal fibrosis in the report from Taylor et al, which further decreases the compliance of surrounding tissue and available space for stent expansion. This point highlights the need for consideration of pre-existing arterial compression or stenosis prior to stent placement in nearby veins.

Unique to our first patient is the delayed onset and mild severity of arterial symptoms following stent placement, as well as the pursuit of conservative management. In our second case and all prior reports, patients developed severe symptoms requiring arterial stenting either immediately postoperatively or within 1 day.^2-7^ As our first patient was asymptomatic immediately following venous stenting and subsequent imaging was performed 2 weeks later, it is not possible to know exactly when the external iliac artery was occluded. Given the pre-existing compression of the external iliac noted on pre-operative imaging, it is possible that this patient had developed enough prior collateralization to prevent critical ischemia following thrombosis of the external iliac artery.

An additional unique feature of our first patient is that occlusion occurred after stent revision following a period of improved symptoms from the initial stent placement, a characteristic shared only by the patient in the report from Vasudev et al.^7^ This raises the possibility that decreased stent function and deformity over time may be a harbinger of overall decreased regional compliance, and extra care should be taken when planning stent revision.

Finally, balloon-expandable stents were employed in both of our patients, similar to most of the previously reported cases.^2-6^ The high radial force of balloon-expandable stents is desirable in patients with factors that limit tissue elasticity, such as cancer, prior surgery, and radiation. However, this also likely predisposes to increased compressive force on the adjacent artery, and an optimal balance between radial force and flexibility should be further examined for patients with limited room for stent expansion.

## Conclusion

These cases highlight the importance of considering patient-specific factors in the planning of stent placement for venous outflow obstruction to avoid arterial compression, particularly in those with conditions that decrease the surrounding tissue compliance and area available for stent expansion. The 2 cases presented further add to the theme that local mass effect and prior surgery/radiation may predispose to nearby arterial compression following venous stent placement, and finding a balance between stent force and flexibility should be further investigated for patients with these risk factors. Additionally, for patients who have had prior successful venous stenting but present with progressive stent dysfunction, care should be taken during revision, as this may be a sign of worsening overall compliance and increased risk of nearby arterial compression. Our first case is unique as the only reported case with delayed onset of mild symptoms managed conservatively, and further investigation is necessary to examine the relationship between venous stenting and arterial occlusion in patients with pre-existing arterial compression.
